# Association between BMI-discordances in cardiometabolic biomarkers and risk of incident heart failure: a prospective cohort study

**DOI:** 10.1016/j.ajcnut.2026.101399

**Published:** 2026-06-17

**Authors:** Shunming Zhang, Yan Borné, Tao Huang, Le Ma, Lu Qi

**Affiliations:** 1 School of Public Health, Xi’an Jiaotong University Health Science Center, Xi’an, China; 2 Nutritional Epidemiology, Department of Clinical Sciences Malmö, Lund University, Malmö, Sweden; 3 Department of Epidemiology and Biostatistics, School of Public Health, Peking University, Beijing, China; 4 Department of Epidemiology, Celia Scott Weatherhead School of Public Health and Tropical Medicine, Tulane University, New Orleans, LA, United States; 5 Department of Nutrition, Harvard T.H. Chan School of Public Health, Boston, MA, United States

**Keywords:** obesity, BMI, heart failure, cohort study, cardiometabolic biomarkers

## Abstract

**Background:**

Obesity is a major risk factor for heart failure (HF). However, individuals with similar BMI, the main diagnostic measure of obesity, exhibit considerable heterogeneity in developing HF.

**Objectives:**

This study aimed to investigate the association between metabolically discordant subgroups characterized by cardiometabolic biomarkers deviated from those predicted by BMI and risk of incident HF.

**Methods:**

Data from 394,198 participants in the UK Biobank were analyzed. A data-driven cluster approach was used to classify BMI subgroups according to cardiometabolic biomarker profiles. Incident HF during the follow-up was ascertained using the International Classification of Diseases, Tenth Revision codes. Cox proportional hazards models were applied to assess the association of the subclassified BMI with HF risk.

**Results:**

Over a median follow-up of 12.3 y, 5176 (2.94%) males and 3602 (1.65%) females developed the first incidence of HF. Compared with participants in BMI-concordant subgroups, individuals with C-reactive protein or blood glucose deviating from the expected risk based on their BMI showed a higher risk of HF. The fully adjusted hazard ratios (95% confidence intervals) for HF in relation to discordant inflammatory state and discordant hyperglycemic status were 1.59 (1.40, 1.80) and 1.39 (1.22, 1.60) in males and 2.03 (1.77, 2.34) and 1.80 (1.53, 2.12) in females, respectively. In contrast, individuals with discordantly high blood pressure (only in females) or adverse blood lipid levels demonstrated a lower risk of HF than those in BMI-concordant subgroups. No significant association with HF was observed for discordant liver transaminase subgroups.

**Conclusions:**

Metabolically distinct BMI subgroups exhibit varying risks of HF, suggesting that subclassifying BMI based on cardiometabolic biomarkers may facilitate the precision prevention of HF.

## Introduction

Heart failure (HF) is a major contributor to the global burden of cardiovascular disease (CVD), affecting ∼55 million people worldwide [1]. In Western countries, HF is the most common diagnosis for hospitalization in persons ≥65 y [2]. The prevalence of HF continues to grow at an alarming rate, driven by population aging [1]. Although the prognosis of HF has improved significantly, it remains a leading cause of global mortality, morbidity, and reduced quality of life, accompanied by high healthcare utilization and costs [3]. In addition, HF has traditionally been viewed as the end-stage of other cardiovascular conditions, leading to its underprioritization in the cardiovascular health agenda, compared with coronary heart disease and stroke [4]. Therefore, prioritizing the primary prevention of HF has become an urgent clinical and public health imperative [4,5].

Overweight and/or obesity, as defined by BMI, are well-established risk factors of HF [6,7]. However, individuals with similar BMI values do not uniformly exhibit the same risk of HF [8,9]. Consequently, identifying subpopulations whose cardiometabolic risk deviates from what would be expected based on BMI (e.g., metabolically unhealthy normal weight or metabolically healthy obesity) could help inform precision prevention strategies for HF. Recent studies in European populations have utilized a data-driven cluster analysis approach to identify 5 reproducible discordant subgroups, where cardiometabolic biomarkers diverged from those predicted by BMI [10]. The subclassified BMI subgroups were based on various obesity-related cardiometabolic phenotypes, including adiposity, blood pressure, liver and kidney function, inflammation, blood lipids, and blood glucose. Notably, these distinct discordant subgroups showed heterogeneous associations with risks of CVD (only including coronary heart disease and stroke) [10]. Although there are shared prevention targets for coronary heart disease/stroke and HF, the prevention of HF necessitates targeted strategies that address its unique pathophysiology and etiologies [4,6,11]. In addition, findings observed for coronary heart disease and stroke may not be directly generalizable to HF, as metabolic dysfunction and obesity may contribute differently to myocardial remodeling, ventricular dysfunction, and HF development [4]. However, it remains unclear whether similar heterogeneity exists in the relationship between these metabolically discordant subgroups and risk of HF.

To fill this knowledge gap, we investigated the association of subpopulations with BMI-discordant cardiometabolic profiles with risk of incident HF in the UK Biobank. We used previously identified and validated subgroups, including those validated in the UK Biobank population [10,12], rather than deriving new subgroups specifically for the present analysis.

## Methods

### Study population

The UK Biobank is a multicenter, prospective population-based study in the United Kingdom, with >500,000 adults [13]. Eligible participants (inclusion criteria) were aged 37–73 y at recruitment between 2006 and 2010 in 22 assessment centers from England, Scotland, and Wales. At the initial visit, they were invited to provide biological samples, complete a touch-screen questionnaire and a computer-assisted interview, and undergo a physical examination. The study was conducted in accordance with the Declaration of Helsinki. The current study was approved by the North West Multicenter Research Ethics Committee (11/NW/0382), and all participants provided written informed consent.

The inclusion criteria for the present study were that participants had provided written informed consent. Participants were excluded if they had missing data on cardiometabolic biomarkers or smoking status, which may have introduced potential selection bias, or if they had baseline prevalent CVD (including coronary heart disease and stroke) or HF based on self-reported and electronic health records. This manuscript adhered to the STROBE guidelines [14].

### Assessment of cardiometabolic biomarkers

BMI and waist-to-hip ratio (WHR) were derived from baseline anthropometric measurements. Systolic and diastolic blood pressures were obtained using standard automated devices; when unavailable, manual sphygmometers were used as an alternative. Baseline blood samples were used to determine levels of blood glucose, serum creatinine (SCR), alanine aminotransferase (ALT), C-reactive protein (CRP), triglycerides (TG), low-density lipoprotein cholesterol (LDL-C), and high-density lipoprotein cholesterol (HDL-C).

### Ascertainment of incident HF and follow-up

Incident HF cases were ascertained through linkage with hospital inpatient records and death registers. HF was defined according to the International Classification of Diseases, Tenth Revision codes I11.0, I13.0, I13.2, I50.0, I50.1, and I50.9 [15]. Participants were followed up from the date of recruitment until the date of the first occurrence of HF, death, loss to follow-up, or the following end dates: 30 September, 2021 for England, 28 February, 2018 for Wales, and 31 July, 2021 for Scotland.

### Assessment of covariates

Baseline information on age (continuous: years), sex (male or female), ethnicity (White or non-White), Townsend deprivation index (continuous), current smoking status (yes or no), alcohol drinking status (never, previous, or current), physical activity (using the International Physical Activity Questionnaire; continuous: metabolic equivalent-min/wk), family history of CVD (yes or no), use of blood pressure medication (yes or no), use of cholesterol-lowering medication (yes or no), use of insulin (yes or no), and dietary intake was collected using touch-screen questionnaires. To assess overall diet quality, a healthy diet score (continuous: 0–5 points) was calculated using the 5 food groups (vegetables, fruits, fish, unprocessed red meats, and processed meats), as described in our recent work [16].

### Identification of the subclassified BMI subgroups

We classified participants into BMI-subclassified cardiometabolic profiles using the clustering approach described by Coral et al. [10]. Briefly, for each cardiometabolic biomarker (including WHR, systolic blood pressure, diastolic blood pressure, ALT, SCR, CRP, blood glucose, HDL-C, TG, and LDL-C), we fitted sex-specific (accounting for sex-specific differences in cardiometabolic biomarkers and HF epidemiology [17]) age- and smoking-adjusted linear regression models with BMI as the independent variable. The residuals from these models (observed minus BMI-predicted values) were extracted, standardized, and used as inputs to a partitioning clustering algorithm [10,18]. Six previously validated profiles were selected, including 1 concordant and 5 discordant profiles [10,12]. Each participant was assigned to the profile with the highest membership probability, consistent with recent studies [12,18]. The profiles (subgroups) were: baseline concordant, with cardiometabolic marker values aligned with BMI-predicted ranges; discordant hypertensive, characterized by higher-than-expected blood pressure (females only); discordant adverse lipid, characterized by higher TG and LDL-C but lower HDL-C; discordant liver transaminase, with higher ALT; discordant inflammatory state, with higher CRP; and discordant hyperglycemic, with higher blood glucose (Supplemental Figure 1). Consistent with previous studies [10,12], discordant hypertensive clusters were identified only in females, which may be related to sex-specific hormonal and life-course factors [19].

### Statistical analysis

Categorical variables were presented as frequencies (percentages) and continuous variables as means ± SDs for descriptive purposes. Three Cox proportional hazards models were fitted to assess the risk of incident HF associated with the discordant profiles, with the concordant profile as the reference group. Using Schoenfeld residuals, no violation of the proportional hazards assumption was found. The first model was adjusted for age, ethnicity, and Townsend deprivation index. The second model was further adjusted for current smoking status, alcohol drinking status, physical activity, healthy diet score, and family history of CVD. The final model was additionally adjusted for the use of blood pressure medication, use of cholesterol-lowering medication, and use of insulin. Missing covariate data were handled by multiple imputation. Furthermore, to better understand how HF risk may change over time in the different clusters and provide information about the absolute risks, we used inverse probability weights for covariate adjustment to plot adjusted survival curves [20,21].

To assess the robustness of the results, we conducted several sensitivity analyses. First, we estimated hazard ratios (HRs) and 95% confidence intervals (CIs) for a 10% shift in membership probability from the baseline concordant profile to each discordant profile, rather than assigning participants to the maximum-probability cluster as in the primary analysis. Second, to minimize potential reverse causation, we repeated the analyses after excluding participants who developed HF within the first 2 y of follow-up. Third, we fitted Fine-Gray subdistribution hazard regression models to account for death as a competing risk. Finally, because individuals with a history of myocardial infarction are at higher risk of developing HF, we used HF without prior myocardial infarction as the outcome, censoring any HF events that occurred after a myocardial infarction, as done in our previous work [22].

All statistical analyses were performed with SAS version 9.4 (SAS Institute Inc.) and R version 4.4.2 (The R Foundation for Statistical Computing). Two-sided *P* values <0.05 were deemed statistically significant.

## Results

After applying all the inclusion and exclusion criteria, a total of 394,198 individuals were included in the final analysis (Supplemental Figure 2). During a median follow-up of 12.3 (IQR: 11.4, 13.1) y, a total of 8778 HF onset cases (including 5176 males and 3602 females) were documented.

### Baseline characteristics of study participants

Table 1 shows the baseline characteristics of study participants overall and according to whether they developed incident HF during follow-up. The total participants had a mean ± SD of age 56.2 ± 8.1 y, with 44.7% being males. Participants who developed HF were older, were more likely to be males, had a higher Townsend deprivation index, and tended to have adverse cardiometabolic risk factors compared with those without incident HF. They were also more likely to engage in unhealthy behaviors (e.g., smoking and physical inactivity), report a family history of CVD, and use blood pressure medication, cholesterol-lowering medication, or insulin. In addition, the participant characteristics across the distinct subclassified BMI clusters are shown in Supplemental Table 1 for males and Supplemental Table 2 for females. In both sexes, individuals in the discordant hypertensive (in females) and discordant adverse lipid subgroups had lower usage rates of blood pressure medication, cholesterol-lowering medication, and insulin than those in other discordant subgroups. In addition, participants in the discordant hyperglycemic cluster had the highest usage rates of antihypertensive medication, cholesterol-lowering medications, and insulin.

**TABLE 1:** Baseline characteristics of study participants^1^.

Characteristics	Total	Incident heart failure	
		No	Yes
Number of participants	394,198	385,420	8778
Age (y)	56.2 ± 8.1	56.1 ± 8.1	61.8 ± 6.4
Sex			
Male	176,103 (44.7)	170,927 (44.4)	5176 (59.0)
Female	218,095 (55.3)	214,493 (55.6)	3602 (41.0)
Ethnicity			
White	372,377 (94.5)	364,032 (94.5)	8345 (95.1)
Non-White	16,977 (4.3)	16,645 (4.3)	332 (3.8)
Missing	4844 (1.2)	4743 (1.2)	101 (1.2)
Townsend deprivation index	–1.37 ± 3.05	–1.38 ± 3.04	–0.89 ± 3.28
BMI (kg/m^2^)	27.3 ± 4.7	27.2 ± 4.7	29.6 ± 5.8
Waist circumference (cm)	89.8 ± 13.3	89.6 ± 13.2	98.0 ± 14.8
Waist-to-hip ratio	0.87 ± 0.09	0.87 ± 0.09	0.92 ± 0.09
Systolic blood pressure (mmHg)	137.9 ± 18.6	137.7 ± 18.6	145.9 ± 19.6
Diastolic blood pressure (mmHg)	82.5 ± 10.1	82.5 ± 10.1	84.0 ± 10.7
Blood glucose (mmol/L)	5.09 ± 1.17	5.08 ± 1.14	5.50 ± 1.94
Serum creatinine (umol/L)	71.8 ± 17.1	71.6 ± 16.3	77.9 ± 37.6
Alanine aminotransferase (U/L)	23.4 ± 14.1	23.3 ± 14.1	24.5 ± 15.5
C-reactive protein (mg/L)	2.55 ± 4.26	2.52 ± 4.20	4.04 ± 6.04
High-density lipoprotein cholesterol (mmol/L)	1.46 ± 0.38	1.46 ± 0.38	1.37 ± 0.38
Low-density lipoprotein cholesterol (mmol/L)	3.61 ± 0.85	3.62 ± 0.85	3.47 ± 0.91
Triglycerides (mmol/L)	1.73 ± 1.01	1.73 ± 1.01	1.92 ± 1.10
Current smoking status			
Yes	40,830 (10.4)	39,486 (10.2)	1344 (15.3)
No	353,368 (89.6)	345,934 (89.8)	7434 (84.7)
Alcohol drinking status			
Never	16,731 (4.2)	16,264 (4.2)	467 (5.3)
Previous	13,163 (3.3)	12,673 (3.3)	490 (5.6)
Current	363,863 (92.3)	356,065 (92.4)	7798 (88.8)
Missing	441 (0.1)	418 (0.1)	23 (0.3)
Physical activity (metabolic equivalent-min/wk)	2668.7 ± 2717.6	2670.7 ± 2716.5	2576.2 ± 2767.4
Healthy diet score	3.43 ± 1.17	3.43 ± 1.17	3.30 ± 1.18
Family history of cardiovascular disease	218,090 (55.3)	212,680 (55.2)	5410 (61.6)
Use of blood pressure medication	69,606 (17.7)	66,020 (17.1)	3586 (40.9)
Use of cholesterol-lowering medication	51,833 (13.2)	49,315 (12.8)	2518 (28.7)
Use of insulin	3413 (0.9)	3113 (0.8)	300 (3.4)

1 Data are shown as mean ± SD for continuous variables or as frequency (%) for categorical variables.

### Associations between different subclassified BMI clusters and risk of HF

**FIGURE 1:**
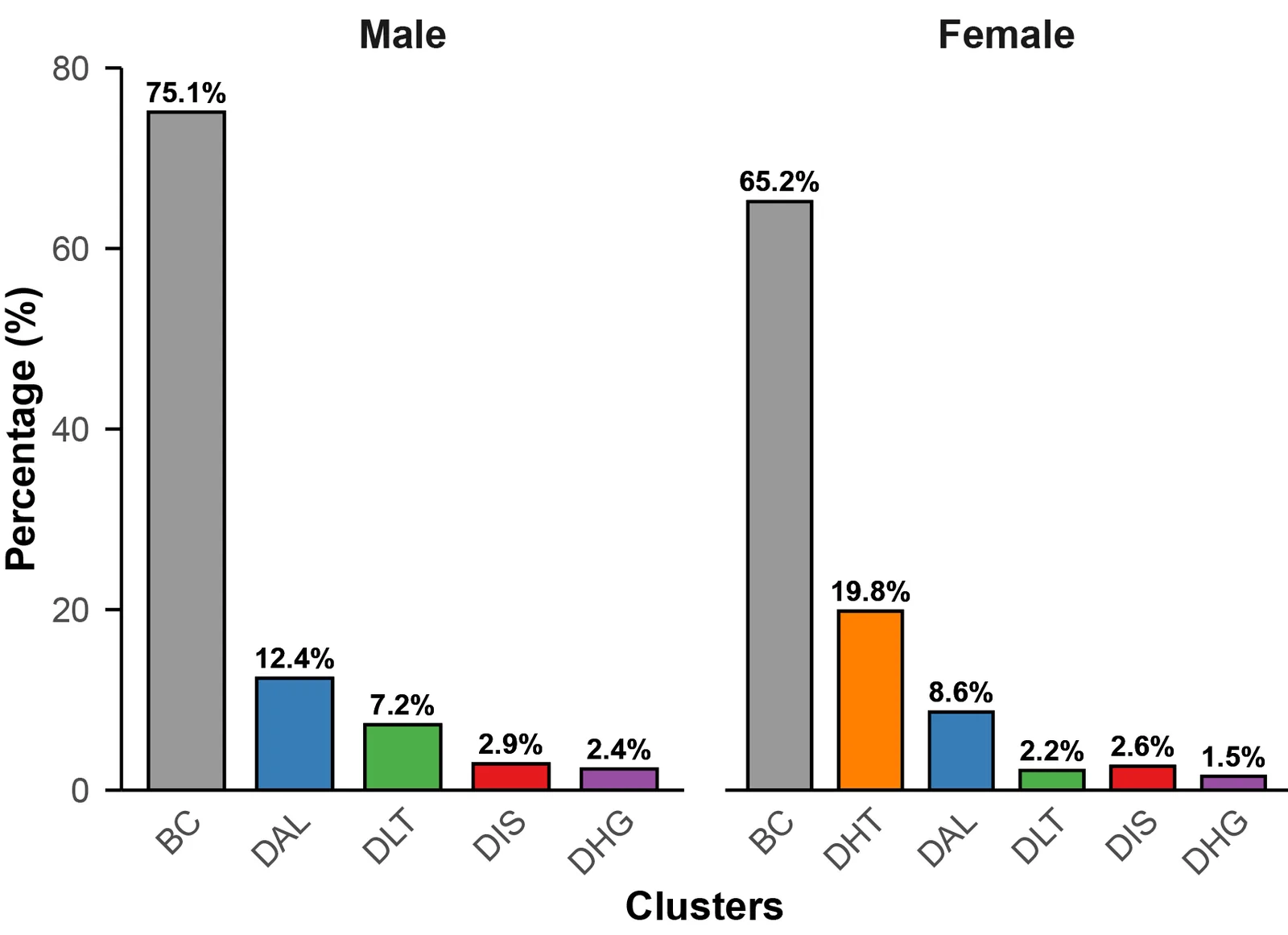
The proportions of cluster allocation. BC, baseline concordant; DAL, discordant adverse lipid; DHG, discordant hyperglycemic; DHT, discordant hypertensive; DIS, discordant inflammatory state; DLT, discordant liver transaminase.

**FIGURE 2:**
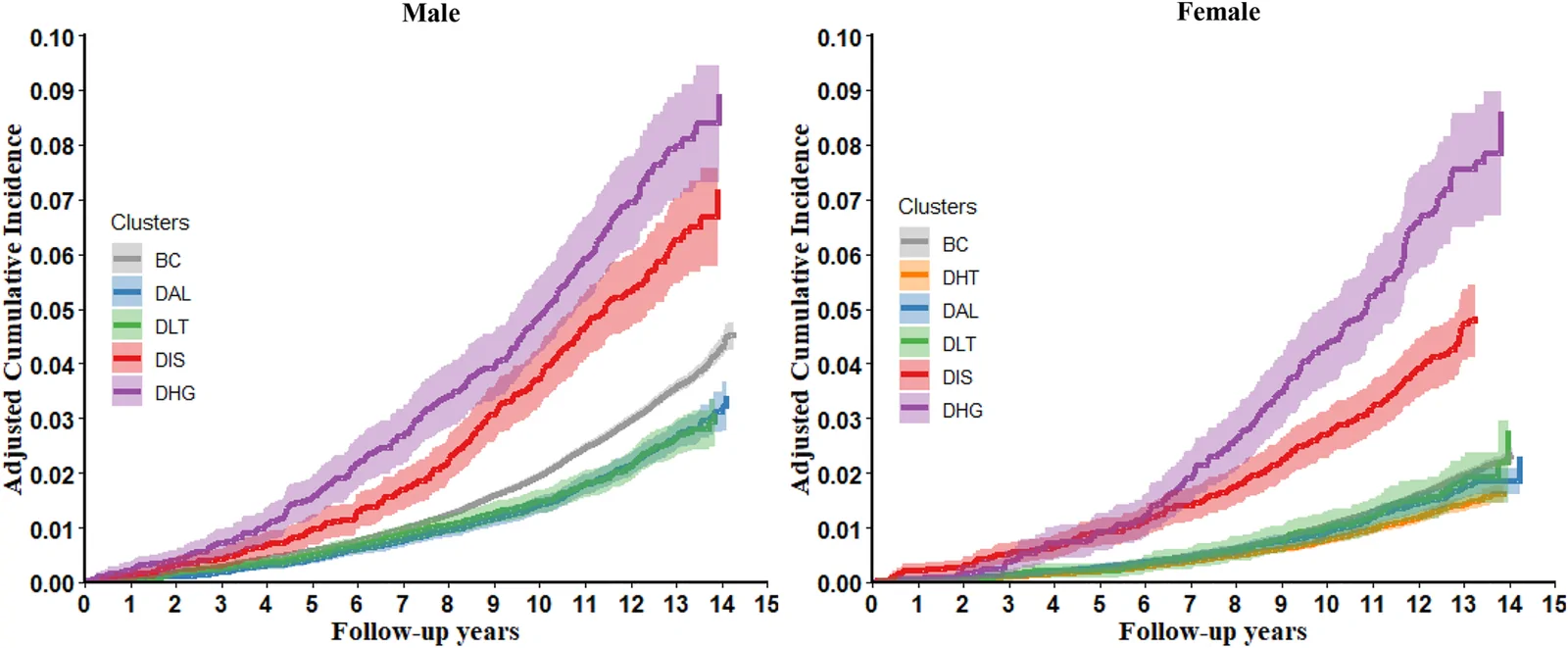
Adjusted cumulative incidence of heart failure across the different clusters. The curves were estimated using inverse probability weights and adjusted for age, ethnicity, Townsend deprivation index, current smoking status, alcohol drinking status, physical activity, healthy diet score, family history of cardiovascular disease, use of blood pressure medication, use of cholesterol-lowering medication, and use of insulin. Solid lines represent the adjusted cumulative incidence, and shaded areas indicate the 95% confidence intervals. BC, baseline concordant; DAL, discordant adverse lipid; DHG, discordant hyperglycemic; DHT, discordant hypertensive; DIS, discordant inflammatory state; DLT, discordant liver transaminase.

The prevalence of the 6 different clusters in males and females was 75.1% and 65.2% for the baseline concordant cluster, 19.8% for the discordant hypertensive cluster (only identified in females), 12.4% and 8.6% for the discordant adverse lipid cluster, 7.2% and 2.2% for the discordant liver transaminase cluster, 2.9% and 2.6% for the discordant inflammatory state cluster, and 2.4% and 1.5% for the discordant hyperglycemic cluster (Figure 1). Table 2 presents the association between different clusters and the risk of incident HF. After adjusting for socioeconomic characteristics, lifestyle factors, family history of CVD, and use of medications, the discordant hypertensive cluster only in females (HR: 0.89; 95% CI: 0.81, 0.98) and the discordant adverse lipid cluster (male HR: 0.82; 95% CI: 0.75, 0.91; female HR: 0.88; 95% CI: 0.77, 0.99) were significantly associated with lower risk of incident HF. In contrast, the discordant inflammatory state cluster and the discordant hyperglycemic cluster were significantly associated with a higher risk of incident HF. For the discordant inflammatory state cluster, the fully adjusted HRs (95% CIs) were 1.59 (1.40, 1.80) in males and 2.03 (1.77, 2.34) in females. For the discordant hyperglycemic cluster, the corresponding HRs (95% CIs) were 1.39 (1.22, 1.60) and 1.80 (1.53, 2.12), respectively. No significant association was observed for the discordant liver transaminase cluster in either males or females. Moreover, participants in the discordant inflammatory state and discordant hyperglycemic profiles had a higher absolute risk of incident HF than those in the baseline concordant profile. In contrast, the discordant hypertensive, discordant adverse lipid, and discordant liver transaminase profiles were associated with lower absolute risk relative to the baseline concordant profile (Figure 2).

**TABLE 2:** Association between different subclassified BMI clusters and risk of incident heart failure^1^.

	Clusters					
	BC	DHT	DAL	DLT	DIS	DHG
Male						
Number of participants	132,256	—	21,820	12,756	5126	4145
Number of cases	3885		470	280	261	280
Person-years	1,536,055	—	255,534	148,323	57,236	46,915
Incidence per 1000 person-years	2.53	—	1.84	1.89	4.56	5.97
Model 1	1.00 (reference)	—	0.80 (0.73, 0.88)	0.91 (0.81, 1.03)	1.68 (1.48, 1.90)	2.09 (1.85, 2.36)
Model 2	1.00 (reference)	—	0.79 (0.72, 0.87)	0.91 (0.80, 1.03)	1.60 (1.41, 1.81)	2.02 (1.79, 2.29)
Model 3	1.00 (reference)		0.82 (0.75, 0.91)	0.91 (0.81, 1.03)	1.59 (1.40, 1.80)	1.39 (1.22, 1.60)
Female						
Number of participants	142,178	43,249	18,833	4751	5758	3326
Number of cases	2306	528	272	74	220	202
Person-years	1,688,372	510,841	224,247	56,470	66,807	39,000
Incidence per 1000 person-years	1.37	1.03	1.21	1.31	3.29	5.18
Model 1	1.00 (reference)	0.81 (0.74, 0.89)	0.88 (0.77, 0.99)	1.06 (0.84, 1.34)	2.22 (1.93, 2.55)	3.06 (2.65, 3.54)
Model 2	1.00 (reference)	0.84 (0.77, 0.93)	0.85 (0.75, 0.97)	1.06 (0.84, 1.34)	2.09 (1.82, 2.40)	2.88 (2.50, 3.33)
Model 3	1.00 (reference)	0.89 (0.81, 0.98)	0.88 (0.77, 0.99)	1.01 (0.80, 1.28)	2.03 (1.77, 2.34)	1.80 (1.53, 2.12)

Model 1 was adjusted for age, ethnicity, and Townsend deprivation index.

Model 2 was further adjusted for current smoking status, alcohol drinking status, physical activity, healthy diet score, and family history of cardiovascular disease.

Model 3 was additionally adjusted for use of blood pressure medication, use of cholesterol-lowering medication, and use of insulin.

Abbreviations: BC, baseline concordant; DAL, discordant adverse lipid; DHG, discordant hyperglycemic; DHT, discordant hypertensive; DIS, discordant inflammatory state; DLT, discordant liver transaminase.

1 Values are hazard ratios (95% confidence intervals) derived from the Cox proportional hazards model unless otherwise indicated.

### Sensitivity analyses

**FIGURE 3:**
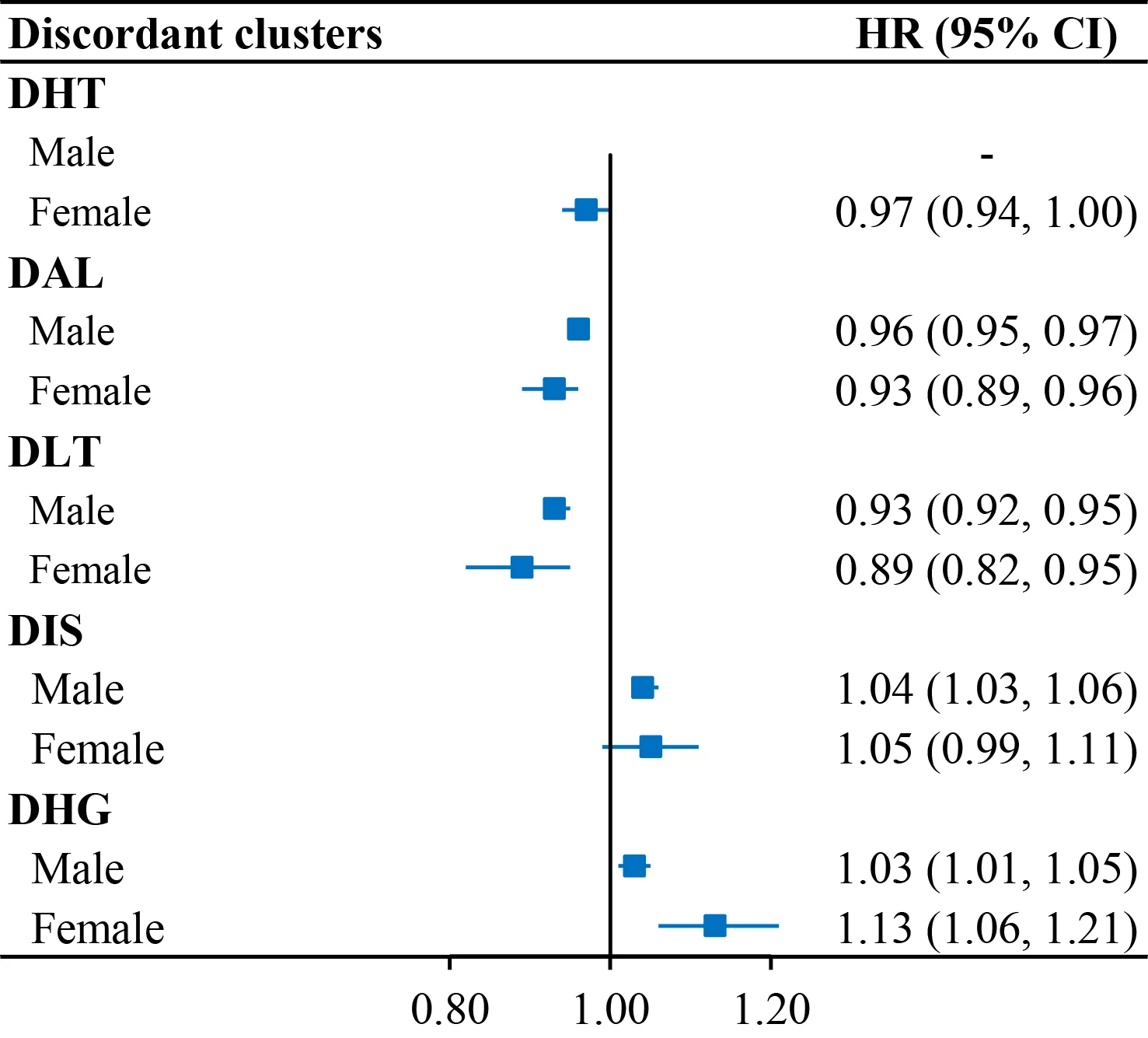
Association of different clusters with risk of incident heart failure, estimating effects shifting 10% probability from the baseline concordant profile to each of the discordant profiles. Cox proportional hazards models were adjusted for age, ethnicity, Townsend deprivation index, current smoking status, alcohol drinking status, physical activity, healthy diet score, family history of cardiovascular disease, use of blood pressure medication, use of cholesterol-lowering medication, and use of insulin. Sample sizes of events/total are 5176/176,103 in males and 3602/218,095 in females. CI, confidence interval; DAL, discordant adverse lipid; DHG, discordant hyperglycemic; DHT, discordant hypertensive; DIS, discordant inflammatory state; DLT, discordant liver transaminase; HR, hazard ratio.

**FIGURE 4:**
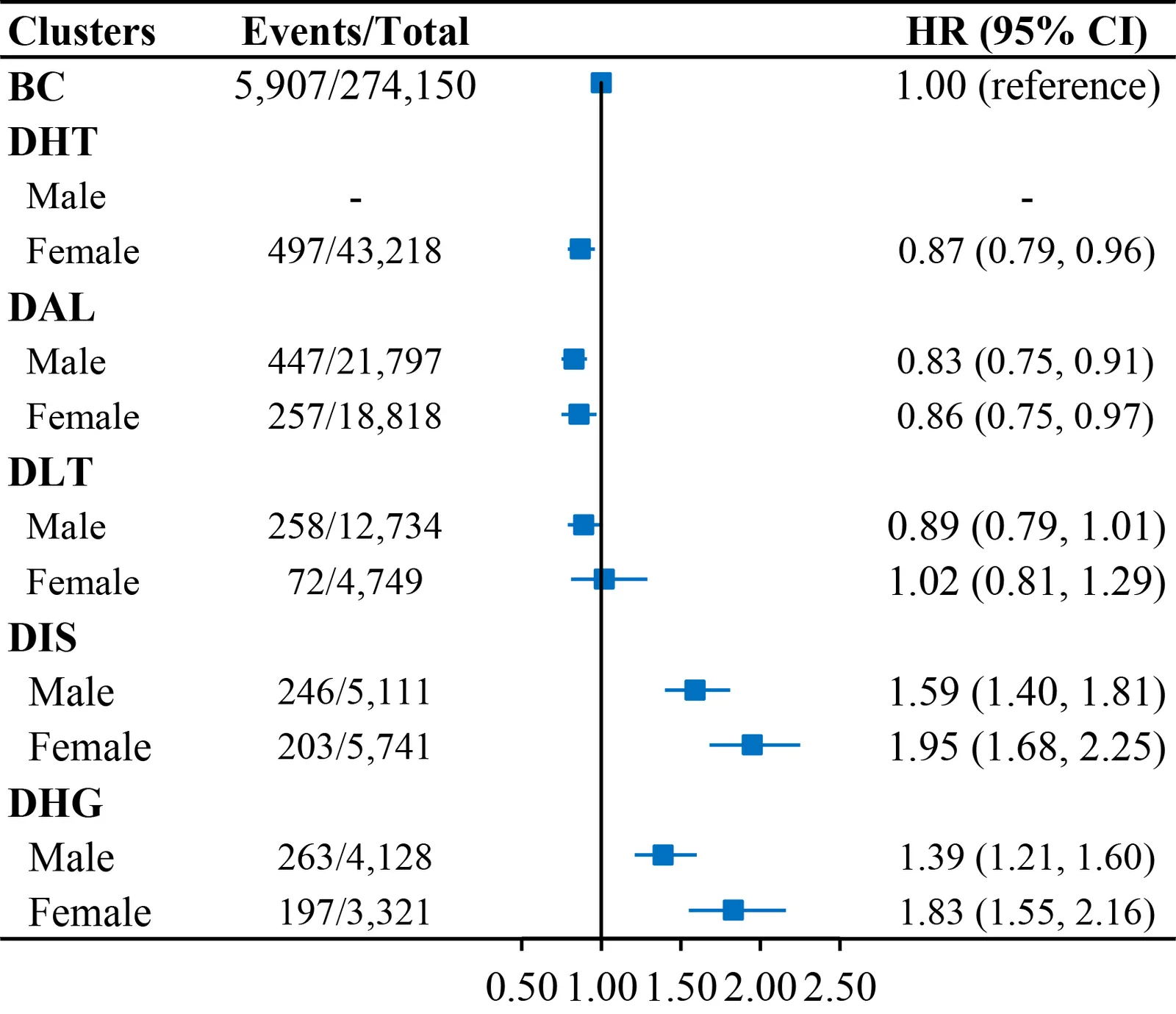
Association of different clusters with risk of incident heart failure, excluding heart failure cases that occurred within the first 2 y of follow-up. Cox proportional hazards models were adjusted for age, ethnicity, Townsend deprivation index, current smoking status, alcohol drinking status, physical activity, healthy diet score, family history of cardiovascular disease, use of blood pressure medication, use of cholesterol-lowering medication, and use of insulin. BC, baseline concordant; CI, confidence interval; DAL, discordant adverse lipid; DHG, discordant hyperglycemic; DHT, discordant hypertensive; DIS, discordant inflammatory state; DLT, discordant liver transaminase; HR, hazard ratio.

When shifting 10% probability from the baseline concordant profile to the discordant profiles instead of assigning participants to a specific cluster based on the maximal profile probability, the associations between the discordant profiles and HF risk remained essentially unchanged (Figure 3). For example, a 10% higher discordant hyperglycemic cluster probability was significantly associated with higher risks of HF (male HR: 1.03; 95% CI: 1.01, 1.05; female HR: 1.13; 95% CI: 1.06, 1.21). When models were rerun after excluding participants with HF that occurred within the first 2 y of follow-up, the results were consistent with those of primary analyses (Figure 4). Furthermore, the association between different clusters and risk of HF was robust when accounting for the competing risk of death (Supplemental Figure 3) or using HF without prior myocardial infarction as the outcome (Supplemental Figure 4).

## Discussion

In this large population cohort, we observed that relative to the BMI-concordant subgroup, participants in the discordant inflammatory state and discordant hyperglycemic subgroups had a higher risk of incident HF. In contrast, the discordant hypertensive (observed only in females) and discordant adverse lipid subgroups were associated with a lower HF risk, whereas the discordant liver transaminase subgroup was not associated with HF risk.

As expected, our results indicated that BMI-discordant cardiometabolic clusters showed divergent risks of HF, supported by recent evidence that BMI alone may not adequately capture individual health risk [[23], [24], [25], [26]]. Although excess adiposity contributes to HF through hemodynamic and structural mechanisms, the substantial variation in HF risk among individuals with similar BMI suggests that additional cardiometabolic pathways are involved [27]. These pathways may include adipose tissue dysfunction, particularly visceral fat expansion and inflammatory remodeling, which can promote adverse cardiac remodeling and dysfunction through altered adipokine signaling [28]. Our findings underscore the importance of cardiometabolic phenotyping for earlier risk identification and more targeted HF prevention.

The discordant inflammatory state subgroup was associated with an increased risk of HF. Several previous studies have indicated a positive association between CRP and the risk of HF [29,30]. Similarly, this subgroup was associated with a higher risk of major adverse cardiovascular events (excluding HF) compared with the concordant subgroup [10]. Our study further explores the heterogeneous associations between subclassified BMI phenotypes and the risk of HF, thereby extending the understanding of BMI-related cardiovascular disease risk profiles. Individuals with inflammation levels exceeding those predicted by BMI may therefore represent a phenotype in which inflammatory burden, rather than excess adiposity per se, predominates in driving myocardial injury and subsequent HF. Compelling evidence emphasizes the clinical and pathophysiological significance of inflammation in the early development of HF [31], lending further support to our findings. Moreover, recent research has demonstrated that vaccine therapy against the inflammatory cytokine may be useful to prevent the onset of HF [32], an effect that could be particularly beneficial for individuals in a pro-inflammatory metabolic state.

Likewise, we found that the discordant hyperglycemic subgroup was associated with a higher risk of HF. In contrast, Coral et al. [10] reported that the subgroup was associated with a lower incidence of major adverse cardiovascular events (excluding HF). This discrepancy may be due to the underlying pathophysiology differences between atherosclerotic CVD and HF. Specifically, elevated blood glucose levels may initiate a cascade of inflammatory responses, endothelial dysfunction, and microvascular impairment [33], all of which can compromise myocardial relaxation. Moreover, a hyperglycemic metabolic state may further exacerbate myocardial dysfunction, thereby contributing to the development of HF. In addition, higher blood glucose has been associated with increased risk of HF in previous studies [34], lending further support to our findings.

Interestingly, both the discordant hypertensive and discordant adverse lipid subgroups were associated with a lower risk of HF. In a study by Coral et al. [10], individuals in these 2 subgroups exhibited a lower prevalence of coronary heart disease, which partially supports our findings. Of note, the discordant hypertensive subgroup was characterized not only by BMI-discordant higher blood pressure but also by BMI-discordant lower WHR and higher HDL-C. This may partly explain its inverse association with HF risk. Similarly, individuals in the discordant adverse lipid subgroup had lower blood glucose at baseline. Taken together, the inverse associations observed for the discordant blood pressure and lipid subgroups should be interpreted with caution. These findings do not indicate that elevated blood pressure or adverse lipid profiles are protective against HF. Rather, these discordant clusters reflect biomarker levels higher than predicted by BMI, rather than absolute hypertension or dyslipidemia. In addition, we cannot exclude the possibility that these findings occurred by chance.

Furthermore, the discordant liver transaminase subgroup was not associated with risk of HF. This finding suggests that individuals with BMI-discordant elevations in ALT have HF risk comparable to that of BMI-concordant individuals, despite having elevated levels of a biomarker typically considered adverse. In contrast, the study by Coral et al. [10] reported that this discordant subgroup was associated with a lower incidence of major adverse cardiovascular events. Notably, previous studies indicated that ALT levels in the normal range displayed an inverse association with CVD [35,36]. In addition, a recent study indicated that lean metabolic dysfunction-associated steatotic liver disease, defined as BMI <23/25 kg/m^2^ in the Chinese cohorts/the UK Biobank, was associated with higher ALT levels but a lower risk of CVD (including HF) compared with its nonlean counterpart [37]. Taken together, these findings suggest that the relationship between BMI-discordant liver enzymes and CVD risk is more complex than is generally appreciated.

From a clinical perspective, our findings should not be interpreted as a call to modify current guideline-based management of cardiometabolic risk factors. Instead, these findings underscore the heterogeneity of cardiometabolic risk within BMI categories and highlight the limitation of relying on BMI alone for HF risk assessment. Although the proportion of individuals with markedly discordant inflammatory or glycemic profiles was relatively small, these subgroups accounted for a disproportionate burden of HF risk. Thus, identifying individuals with BMI-discordant inflammatory or glycemic phenotypes may improve risk stratification and inform future research into targeted preventive strategies for HF. In particular, incorporating routinely available biomarkers, such as CRP and blood glucose, into risk assessment among individuals with similar BMI may help identify those at higher residual risk of HF. These findings may also guide future trial enrichment strategies and support more personalized risk communication, although changes to guideline-based management should await confirmation from prospective interventional studies.

To the best of our knowledge, this is the first study examining the association between the subclassified BMI based on cardiometabolic profiles and risk of HF. A major strength of this study is access to the large samples the UK Biobank affords, including the long follow-up and detailed data on cardiometabolic biomarkers. In addition, a series of sensitivity analyses validated the reliability of our results. More importantly, the use of clustering techniques to capture BMI-discordant subgroups adds a novel dimension to risk stratification of HF.

Nonetheless, several limitations should be considered. First, cardiometabolic biomarkers were measured at recruitment, and potential changes during follow-up cannot be captured. However, the ability of baseline biomarkers to predict future HF risk remains of public health relevance. Second, although BMI-biomarker associations may be nonlinear, linear regression was used to define simple, interpretable discordance residuals. Third, the clustering approach is typically applied at the population level, which limits its direct applicability to individual-level risk prediction. Fourth, although we adjusted for a comprehensive range of covariates, residual confounding (e.g., glucose-lowering therapies other than insulin) cannot be entirely excluded, and causality cannot be established. However, the consistency of the results across multiple sensitivity analyses reinforces the robustness of our findings. Finally, because the participants were predominantly of European ancestry and middle-aged or older adults, the generalizability of these results to other populations may be limited.

In conclusion, our study demonstrates that metabolically distinct BMI subgroups exhibit heterogeneous risks of HF. Specifically, individuals with disproportionately elevated inflammation markers or blood glucose levels relative to their BMI exhibit a higher risk of HF than those with BMI-concordant cardiometabolic profiles. In contrast, subpopulations with other BMI-discordant cardiometabolic profiles showed inverse or null associations with HF risk. These findings may inform personalized BMI management strategies to optimize early prevention of HF.

## Author contributions

The authors’ responsibilities were as follows – SZ, LQ: conceived and designed the research; SZ: analyzed the data and wrote the paper; SZ, YB, TH, LM, LQ: revised the manuscript critically for important intellectual content; and all authors: read and approved the final manuscript.

## Data availability

The data that support the findings of this study are available on application to the UK Biobank (application number 44430).

## Declaration of Generative AI and AI-assisted technologies in the writing process

The authors declare that no generative AI or AI-assisted technologies were used in the writing of this manuscript.

## Funding

This work was supported by grants from the Fundamental Research Funds for the Central Universities (No. xzy012024141), Young Elite Scientists Sponsorship Program by CAST (No. 2023QNRC001), China Postdoctoral Science Foundation (No. 2023M732772), and Shaanxi Province Postdoctoral Science Foundation (No. 2023BSHEDZZ19). The study funding sponsor had no role in the design or conduct of the study; the collection, analysis, or interpretation of the data; or the preparation, review, or approval of the manuscript.

## Conflict of interest

The authors report no conflicts of interest.
