# Supplementary material for: Association between BMI-discordances in cardiometabolic biomarkers and risk of incident heart failure: a prospective cohort study
Source: Am J Clin Nutr. 2026 Jun 17;124(2):101399. doi: 10.1016/j.ajcnut.2026.101399 (PMC13494079; doi:10.1016/j.ajcnut.2026.101399)
Supplement: Supplementary file 1 [file mmc1.docx]

**Online Supplementary Materials**

**Title:** Association between BMI-discordances in cardiometabolic biomarkers and risk of incident heart failure: a prospective cohort study

**Author Names**: Shunming Zhang, Yan Borné, Tao Huang, Le Ma, Lu Qi

| Page 2: | **Figure S1.** Profile centers for the distinct clusters |
| --- | --- |
| Page 3: | **Figure S2.** Flow diagram of participant selection |
| Page 4: | **Figure S3.** Association of different clusters with risk of incident heart failure, using Fine-Gray subdistribution hazard models that accounted for the competing risk of death |
| Page 5: | **Figure S4.** Association of different clusters with risk of incident heart failure without prior myocardial infarction |
| Pages 6-7: | **Table S1.** Baseline characteristics of study participants across the validated clusters in males |
| Pages 8-9: | **Table S2.** Baseline characteristics of study participants across the validated clusters in females |

**Figure S1.** Profile centers for the distinct clusters. Abbreviations: BC, baseline concordant; ALT, alanine aminotransferase; CRP, C-reactive protein; DAL, discordant adverse lipid; DBP, diastolic blood pressure; DHG, discordant hyperglycemic; DHT, discordant hypertensive; DIS, discordant inflammatory state; DLT, discordant liver transaminase; HDL-C, high-density lipoprotein cholesterol; LDL-C, low-density lipoprotein cholesterol; SBP, systolic blood pressure; SCR, serum creatinine; SD, standard deviation; TG, triglycerides; WHR, waist-to-hip ratio.

**Figure S2.** Flow diagram of participant selection.

**Figure S3.** Association of different clusters with risk of incident heart failure, using Fine-Gray subdistribution hazard models that accounted for the competing risk of death. Models were adjusted for age, ethnicity, Townsend deprivation index, current smoking status, alcohol drinking status, physical activity, healthy diet score, family history of cardiovascular disease, use of blood pressure medication, use of cholesterol-lowering medication, and use of insulin. Deaths denote the number of deaths that occurred before the onset of incident heart failure. Abbreviations: BC, baseline concordant; CI, confidence interval; DAL, discordant adverse lipid; DHG, discordant hyperglycemic; DHT, discordant hypertensive; DIS, discordant inflammatory state; DLT, discordant liver transaminase; SHR, subdistribution hazard ratio.

**Figure S4.** Association of different clusters with risk of incident heart failure without prior myocardial infarction. Cox proportional hazards models were adjusted for age, ethnicity, Townsend deprivation index, current smoking status, alcohol drinking status, physical activity, healthy diet score, family history of cardiovascular disease, use of blood pressure medication, use of cholesterol-lowering medication, and use of insulin. Abbreviations: BC, baseline concordant; CI, confidence interval; DAL, discordant adverse lipid; DHG, discordant hyperglycemic; DHT, discordant hypertensive; DIS, discordant inflammatory state; DLT, discordant liver transaminase; HR, hazard ratio.

| **Table S1.** Baseline characteristics of study participants across the validated clusters in males ^1^ | | | | | | |  |
| --- | --- | --- | --- | --- | --- | --- | --- |
| Characteristics | Total | Clusters | | | | |  |
|  |  | BC | DAL | DLT | DIS | DHG |  |
| Number of participants | 176,103 | 132,256 | 21,820 | 12,756 | 5,126 | 4,145 |  |
| Age (years) | 56.2±8.2 | 56.4±8.3 | 55.4±8.0 | 54.4±7.9 | 57.3±8.3 | 58.0±7.5 |  |
| Ethnicity |  |  |  |  |  |  |  |
| White | 166,108 (94.3) | 124,878 (94.4) | 20,647 (94.6) | 12,045 (94.4) | 4,862 (94.9) | 3,676 (88.7) |  |
| Non-White | 7,755 (4.4) | 5,739 (4.3) | 908 (4.2) | 529 (4.2) | 193 (3.8) | 386 (9.3) |  |
| Missing | 2,240 (1.3) | 1639 (1.2) | 265 (1.2) | 182 (1.4) | 71 (1.4) | 83 (2.0) |  |
| Townsend deprivation index | -1.34±3.10 | -1.36±3.09 | -1.42±3.05 | -1.34±3.11 | -0.93±3.34 | -0.83±3.34 |  |
| Body mass index (kg/m^2^) | 27.7±4.2 | 27.5±4.2 | 27.7±3.3 | 28.5±3.7 | 28.0±4.8 | 30.3±5.3 |  |
| Waist circumference (cm) | 96.5±11.1 | 95.8±11.3 | 97.2±8.7 | 99.2±9.7 | 98.1±12.4 | 104.5±13.4 |  |
| Waist-to-hip ratio | 0.93±0.06 | 0.93±0.07 | 0.94±0.05 | 0.95±0.05 | 0.95±0.07 | 0.98±0.07 |  |
| Systolic blood pressure (mmHg) | 141.2±17.3 | 140.5±17.1 | 141.5±16.8 | 147.0±18.5 | 140.2±19.6 | 142.7±17.0 |  |
| Diastolic blood pressure (mmHg) | 84.5±9.9 | 83.8±9.5 | 85.8±9.8 | 90.6±11.2 | 83.9±11.4 | 82.8±10.0 |  |
| Blood glucose (mmol/L) | 5.15±1.32 | 5.02±0.74 | 4.87±0.56 | 5.01±0.63 | 5.00±0.72 | 11.42±3.59 |  |
| Serum creatinine (umol/L) | 81.2±17.1 | 81.8±18.6 | 80.2±10.5 | 79.1±9.9 | 80.1±13.6 | 75.6±14.6 |  |
| Alanine aminotransferase (U/L) | 27.4±15.2 | 24.3±9.5 | 28.2±10.6 | 57.6±29.7 | 23.5±11.4 | 34.6±19.1 |  |
| C-reactive protein (mg/L) | 2.41±4.23 | 1.91±2.12 | 1.59±1.19 | 1.88±1.62 | 20.12±12.48 | 2.63±3.10 |  |
| High-density lipoprotein cholesterol (mmol/L) | 1.29±0.31 | 1.34±0.31 | 1.06±0.17 | 1.26±0.28 | 1.19±0.28 | 1.11±0.27 |  |
| Low-density lipoprotein cholesterol (mmol/L) | 3.56±0.83 | 3.52±0.81 | 3.83±0.70 | 3.96±0.97 | 3.29±0.76 | 2.75±0.85 |  |
| Triglycerides (mmol/L) | 1.97±1.14 | 1.66±0.77 | 3.78±1.37 | 2.13±0.91 | 1.63±0.77 | 2.45±1.51 |  |
| Current smoking status |  |  |  |  |  |  |  |
| Yes | 21,762 (12.4) | 16,227 (12.3) | 2,700 (12.4) | 1,459 (11.4) | 890 (17.4) | 486 (11.7) |  |
| No | 154,341 (87.6) | 116,029 (87.7) | 19,120 (87.6) | 11,297 (88.6) | 4,236 (82.6) | 3,659 (88.3) |  |
| Alcohol drinking status |  |  |  |  |  |  |  |
| Never | 4,635 (2.6) | 3,288 (2.5) | 698 (3.2) | 291 (2.3) | 145 (2.8) | 213 (5.1) |  |
| Previous | 5,676 (3.2) | 4,130 (3.1) | 717 (3.3) | 344 (2.7) | 225 (4.4) | 260 (6.3) |  |
| Current | 165,589 (94.0) | 124,691 (94.3) | 20,386 (93.4) | 12,103 (94.9) | 4,750 (92.7) | 3,659 (88.3) |  |
| Missing | 203 (0.1) | 147 (0.1) | 19 (0.1) | 18 (0.1) | 6 (0.1) | 13 (0.3) |  |
| Physical activity (metabolic equivalent-min/week) | 2837.5±2967.0 | 2939.7±3005.2 | 2578.5±2823.2 | 2524.6±2861.9 | 2553.3±2850.4 | 2212.4±2659.6 |  |
| Healthy diet score | 3.12±1.19 | 3.16±1.19 | 3.03±1.18 | 3.01±1.18 | 3.00±1.19 | 3.08±1.18 |  |
| Family history of cardiovascular disease | 91,964 (52.2) | 68,777 (52.0) | 11,566 (53.0) | 6,701 (52.5) | 2,659 (51.9) | 2,261 (54.6) |  |
| Use of blood pressure medication | 34931 (19.8) | 25,956 (19.6) | 3,396 (15.6) | 2,312 (18.1) | 1,172 (22.9) | 2,095 (50.5) |  |
| Use of cholesterol-lowering medication | 29272 (16.6) | 21,415 (16.2) | 2,654 (12.2) | 1,856 (14.6) | 761 (14.9) | 2,586 (62.4) |  |
| Use of insulin | 1945 (1.1) | 912 (0.7) | 28 (0.1) | 39 (0.3) | 40 (0.8) | 926 (22.3) |  |
| Abbreviations: BC, baseline concordant; DAL, discordant adverse lipid; DHG, discordant hyperglycemic; DIS, discordant inflammatory state; DLT, discordant liver transaminase. | | | | | | |  |
| ^1^ Data are shown as mean ± standard deviation for continuous variables or as frequency (%) for categorical variables. | | | | | | |  |

| **Table S2.** Baseline characteristics of study participants across the validated clusters in females ^1^ | | | | | | | |  |
| --- | --- | --- | --- | --- | --- | --- | --- | --- |
| Characteristics | Total | Clusters | | | | | |  |
|  |  | BC | DHT | DAL | DLT | DIS | DHG |  |
| Number of participants | 218,095 | 142,178 | 43,249 | 18,833 | 4,751 | 5,758 | 3,326 |  |
| Age (years) | 56.2±8.0 | 56.1±8.2 | 56.0±7.7 | 56.7±7.6 | 55.8±7.1 | 56.9±8.1 | 58.2±7.5 |  |
| Ethnicity |  |  |  |  |  |  |  |  |
| White | 206,269 (94.6) | 134,264 (94.4) | 41,377 (95.7) | 17,738 (94.2) | 4,551 (95.8) | 5,400 (93.8) | 2,939 (88.4) |  |
| Non-White | 9,222 (4.2) | 6,167 (4.3) | 1,467 (3.4) | 848 (4.5) | 148 (3.1) | 285 (5.0) | 307 (9.2) |  |
| Missing | 2,604 (1.2) | 1,747 (1.2) | 405 (0.9) | 247 (1.3) | 52 (1.1) | 73 (1.3) | 80 (2.4) |  |
| Townsend deprivation index | -1.40±3.00 | -1.36±3.02 | -1.67±2.87 | -1.36±2.99 | -1.26±3.06 | -0.93±3.25 | -0.72±3.29 |  |
| Body mass index (kg/m^2^) | 27.0±5.1 | 27.0±5.2 | 25.8±4.3 | 27.4±4.0 | 28.7±5.1 | 29.6±6.8 | 30.9±7.0 |  |
| Waist circumference (cm) | 84.4±12.4 | 84.5±12.5 | 79.8±10.2 | 88.6±10.0 | 90.0±12.3 | 90.3±14.8 | 95.8±15.9 |  |
| Waist-to-hip ratio | 0.82±0.07 | 0.82±0.07 | 0.78±0.06 | 0.86±0.06 | 0.85±0.07 | 0.84±0.07 | 0.88±0.08 |  |
| Systolic blood pressure (mmHg) | 135.2±19.2 | 130.8±17.4 | 147.8±19.3 | 138.1±18.7 | 138.2±18.8 | 136.1±18.5 | 139.1±18.3 |  |
| Diastolic blood pressure (mmHg) | 80.8±10.0 | 78.3±9.0 | 87.7±9.2 | 82.9±9.9 | 84.3±10.3 | 81.8±10.2 | 80.3±10.2 |  |
| Blood glucose (mmol/L) | 5.05±1.03 | 4.96±0.69 | 4.93±0.43 | 4.98±0.57 | 5.06±0.76 | 5.05±0.75 | 10.63±3.38 |  |
| Serum creatinine (umol/L) | 64.1±12.7 | 64.9±14.2 | 62.7±8.2 | 62.0±9.2 | 62.4±9.6 | 63.6±11.4 | 62.7±15.5 |  |
| Alanine aminotransferase (U/L) | 20.1±12.1 | 19.1±8.0 | 16.8±4.9 | 21.4±8.3 | 70.5±37.5 | 19.2±8.7 | 25.9±14.8 |  |
| C-reactive protein (mg/L) | 2.67±4.27 | 2.31±2.57 | 1.37±1.25 | 2.27±1.87 | 3.48±3.94 | 20.99±11.10 | 4.17±5.06 |  |
| High-density lipoprotein cholesterol (mmol/L) | 1.60±0.38 | 1.55±0.32 | 1.94±0.37 | 1.28±0.21 | 1.51±0.39 | 1.46±0.36 | 1.37±0.39 |  |
| Low-density lipoprotein cholesterol (mmol/L) | 3.65±0.86 | 3.57±0.84 | 3.68±0.75 | 4.33±0.87 | 3.87±0.95 | 3.48±0.80 | 3.02±0.92 |  |
| Triglycerides (mmol/L) | 1.54±0.85 | 1.43±0.62 | 1.11±0.39 | 3.17±1.13 | 1.80±0.84 | 1.59±0.73 | 2.10±1.22 |  |
| Current smoking status |  |  |  |  |  |  |  |  |
| Yes | 19,068 (8.7) | 12,302 (8.7) | 3,564 (8.2) | 1,882 (10.0) | 369 (7.8) | 654 (11.4) | 297 (8.9) |  |
| No | 199,027 (91.3) | 129,876 (91.4) | 39,685 (91.8) | 16,951 (90.0) | 4,382 (92.2) | 5,104 (88.6) | 30,29 (91.1) |  |
| Alcohol drinking status |  |  |  |  |  |  |  |  |
| Never | 12,096 (5.6) | 8,092 (5.7) | 1,510 (3.5) | 1,404 (7.5) | 219 (4.6) | 452 (7.9) | 419 (12.6) |  |
| Previous | 7,487 (3.4) | 5,153 (3.6) | 813 (1.9) | 799 (4.2) | 191 (4.0) | 321 (5.6) | 210 (6.3) |  |
| Current | 19,8274 (90.9) | 128,786 (90.6) | 40,883 (94.5) | 16,604 (88.2) | 4,337 (91.3) | 4,972 (86.4) | 2,692 (80.9) |  |
| Missing | 238 (0.1) | 147 (0.1) | 43 (0.1) | 26 (0.1) | 4 (0.1) | 13 (0.2) | 5 (0.2) |  |
| Physical activity (metabolic equivalent-min/week) | 2520.6±2468.8 | 2502.5±2461.1 | 2740.9±2534.6 | 2375.1±2409.0 | 2238.5±2399.8 | 2184.1±2300.2 | 2132.2±2360.7 |  |
| Healthy diet score | 3.68±1.09 | 3.67±1.09 | 3.77±1.06 | 3.59±1.11 | 3.58±1.11 | 3.50±1.14 | 3.54±1.13 |  |
| Family history of cardiovascular disease | 126,126 (57.8) | 81,398 (57.3) | 25,113 (58.1) | 11,322 (60.1) | 2,844 (59.9) | 3,414 (59.3) | 2,035 (61.2) |  |
| Use of blood pressure medication | 34,675 (15.9) | 21,811 (15.3) | 5,830 (13.5) | 3,195 (17.0) | 1,005 (21.2) | 1,302 (22.6) | 1,532 (46.1) |  |
| Use of cholesterol-lowering medication | 22,561 (10.3) | 15,593 (11.0) | 2,148 (5.0) | 1,852 (9.8) | 614 (12.9) | 539 (9.4) | 1,815 (54.6) |  |
| Use of insulin | 1,468 (0.7) | 582 (0.4) | 34 (0.1) | 32 (0.2) | 28 (0.6) | 31 (0.5) | 761 (22.9) |  |
| Abbreviations: BC, baseline concordant; DAL, discordant adverse lipid; DHG, discordant hyperglycemic; DHT, discordant hypertensive; DIS, discordant inflammatory state; DLT, discordant liver transaminase. | | | | | | | |  |
|  |  |  |  |  |  |  |  |  |
| ^1^ Data are shown as mean ± standard deviation for continuous variables or as frequency (%) for categorical variables. | | | | | | | |  |
